# Zeolite Socony Mobil-Five Coating on Ti-24 Nb-4 Zr-7.9 Sn Promotes Biocompatibility and Osteogenesis In Vitro and In Vivo

**DOI:** 10.1155/2021/5529368

**Published:** 2021-07-29

**Authors:** Xiaodong Hang, Xiaohan Liu, Ying Hu, Yilai Jiao, Lin Wu

**Affiliations:** ^1^Department of Prosthodontics, School and Hospital of Stomatology, China Medical University, Liaoning Provincial Key Laboratory of Oral Diseases, Shenyang 110002, China; ^2^Department of Pediatric Dentistry, Dalian Stomatological Hospital, Dalian, China; ^3^Shenyang National Laboratory for Materials Science, Institute of Metal Research, Chinese Academy of Sciences, Shenyang, China

## Abstract

The aim of this study was to evaluate the biocompatibility and osteogenic potential of a Zeolite Socony Mobil-5 (ZSM-5) coating on a Ti-24 Nb-4 Zr-7.9 Sn (Ti-2448) surface. ZSM-5-modified Ti-2448 (ZSM-5/Ti-2448) and Ti-2448 (control) groups were employed. The physical and chemical properties of the two types of samples were evaluated by scanning electron microscopy, Fourier-transform infrared spectroscopy, nitrogen adsorption/desorption, and contact angle methods. The surface of the ZSM-5/Ti-2448 was rougher than that of the original Ti-2448, while the contact angle of the ZSM-5/Ti-2448 was smaller than that of Ti-2448. In addition, the ZSM-5/Ti-2448 largely increased the specific surface area and introduced silanol groups. A bone-like apatite layer could be formed on the surface of ZSM-5/Ti-2448 after 14 days of incubation in a simulated body fluid. ZSM-5/Ti-2448 was not cytotoxic. The number and alkaline phosphatase (ALP) activity of osteoblasts on ZSM-5/Ti-2448 were significantly higher than those on Ti-2448 surfaces, obtained *in vitro* using 3-(4,5-dimethylthiazole-2-yl)-2,5-diphenyltetrazolium bromide and ALP activity assays. Few inflammatory cells were observed around ZSM-5/Ti-2448 after insertion into the femurs of Japanese white rabbits after 4, 12, and 26 weeks through hematoxylin–eosin staining. The average gray scale of transforming growth factor-*β*1 (TGF-*β*1) on ZSM-5/Ti-2448 peaked earlier than that on Ti-2448, according to immunohistochemical staining. These results indicate that ZSM-5/Ti-2448 has a good biocompatibility and improved early osteogenic potential compared to a noncoated Ti-2448.

## 1. Introduction

In recent years, the *β*-titanium alloy Ti-24 Nb-4 Zr-7.9 Sn (wt.%)(Ti-2448) developed by the Institute of Metal Research, Chinese Academy of Sciences, (PCT/CN2004/001352), for bone plates and spinal fixation systems has attracted considerable interest because of its biocompatibility [1], excellent mechanical strength, and, particularly, relatively low elastic modulus (42 GPa) [2]. The relative elastic modulus of Ti-2448 alloys is lower than that of Ti (106.4 GPa) and Ti-6Al-4V (120 GPa) alloys, which better matches that of the cortical bone (17–20 GPa) and dentin (12–18.6 GPa).

However, Ti-2448 alloys are bioinert and cannot accelerate the healing of bone tissues. In this regard, a bioactive coating on them has been designed to realize a better bone–material contact and ameliorate other adverse reactions such as the bone resorption effects of Ti ions released from the Ti materials [3]. Zeolites are aluminosilicates with uniform microporous structures, which have been utilized commercially for catalysis and separation processes. Zeolite coatings act as a barrier between the metal and corrosive medium to prevent corrosion. In addition, zeolite coatings have elastic moduli of 30–40 GPa [4], which considerably better match those of a bone than titanium alloys [5]. The low elastic modulus is important for artificial bone materials to avoid the shielding-bone resorption caused by an unbalanced stress distribution between the bone and materials [6]. Zeolite Socony Mobil-5 was invented by the Mobil Oil Company in 1975. Among the family of zeolites, ZSM-5 is highly catalytic and has the potential to catalyze biological molecules within the body. SC79-loaded ZSM-5/chitosan (ZSM-5/CS/SC79) porous scaffolds could enhance stem cell osteogenic differentiation and bone regeneration [7].

In this study, we produced a ZSM-5 coating on a Ti-2448 surface by a hydrothermal synthesis [8, 9]. We investigated the hypothesis that ZSM-5-modified Ti-2448 (ZSM-5/Ti-2448) could provide a surface structure that facilitates the proliferation and differentiation of osteoblasts, which would benefit the bone formation in bone defect sites.

## 2. Materials and Methods

### 2.1. Sample Preparation and Surface Properties

Ti-2448 alloys were provided by the Institute of Metal Research, Chinese Academy of Sciences (Shenyang, China). Disks (diameter: 10 mm, thickness: 1 mm) were used for the cell culture experiments. Cylindrical implants (diameter: 2 mm, length: 6 mm) were used for endosseous implantation. Ti-2448 alloys were regarded as a negative control and substrate. All samples were ground with a 1200-grit SiC sandpaper and cleaned ultrasonically in acetone, ethanol, and deionized water, successively. Finally, a ZSM-5 coating was synthesized on the surface of the alloy through the hydrothermal method [10]. The materials were ultrasonically cleaned, autoclaved (134°C/0.21 MPa), and prepared for use. The surface morphologies of the samples were observed by scanning electron microscopy (SEM, Hitachi S3400N, Japan) and energy-dispersive spectrometry (EDS, Inca, Oxford). The sample surface wettability was analyzed using a contact angle measurement system (VSA 2500 XE, AST Products, Tokyo, Japan). Infrared spectra of the prepared samples were measured using a Fourier-transform infrared (FT-IR) spectroscopy (Thermo Fisher Scientific Nicolet 6700, Madison, WI, USA). The texture properties were evaluated by the nitrogen adsorption/desorption method at 77 K using a Micromeritics 3Flex Adsorption Analyzer (Micromeritics, USA).

### 2.2. Evaluation of the Biomineralization

The ZSM-5/Ti-2448 and Ti-2448 samples were incubated in a simulated body fluid (SBF) for 14 days, and the compositions of bioactive deposits on the surfaces of these samples were analyzed by EDS.

### 2.3. Cell Proliferation and Cytotoxicity

A 3-(4,5-dimethylthiazole-2-yl)-2,5-diphenyltetrazolium bromide (MTT) (Sigma, America) assay was used for proliferation testing. MTT was described for the first time by Beyer and Pyl [11] and utilized by Rusen et al. for cellular bioassays to quantify the cellular growth and cytotoxicity [12]. MC3T3-E1 cells were seeded in a 96-well plate at a density of 1 × 10^4^ cells per well. After 24 h, the culture medium was replaced with 100 *μ*L of the leaching liquor of Ti-2448 (negative control), ZSM-5/Ti-2448, and 0.64% phenol solution (positive control). The cells were incubated for 24, 48, and 72 h. At the endpoint, MTT (0.5 mg/ml) was added to the culture for additional 4 h, and then, the cells were treated with 150 *μ*g/L dimethyl sulfoxide (Sigma, America). The absorbance (*A*) at 550 nm was measured using a microplate reader (Spectra Max M5). The relative growth rate (%) (RGR) was calculated by [*A*] test/−[*A*] negative control × 100%.

The cytotoxicity was classified as follows: RGR (%) ≥ 75: noncytotoxic, RGR (%) = 50–74: mildly cytotoxic, RGR (%) = 25–49: moderately cytotoxic, and RGR (%) ≤ 24: markedly cytotoxic.

### 2.4. Alkaline Phosphatase (ALP) Activity

ALP is an indicator of osteogenic differentiation, bone formation, and matrix mineralization [13]. The activity of ALP was detected to analyze the differentiation of MC3T3-E1 cells on ZSM-5/Ti-2448 and Ti-2448. MC3T3-E1 cells (2 × 10^4^ cells/well) were cultured on Ti-2448 (negative control) and ZSM-5/Ti-2448 in 24-well plates for 1, 3, 5, 7, and 10 days. The cells were lysed in 1.5 M Tris-HCL (pH = 10.2) followed by centrifugation. Aliquots of supernatants were subjected to a total protein assay and ALP activity measurements. The total cellular ALP activity was measured by colorimetry at pH of 10.3 in a 2-amino-2-methyl-1-propanol buffer. The change in absorbance at 520 nm was measured using a microplate reader. The ALP activity was expressed by nanomoles of p-nitrophenol liberated per microgram of total cellular protein.

### 2.5. Endosseous Implantation

Twenty adult male Japanese white rabbits (authorization number: SYXK LIAO 2003-0013) (six months old, mass: 2.0–3.0 kg) were used in this study after two weeks of feeding. All animals were supplied by the Experimental Animals Center of China Medical University. The experiments were carried out under the guideline of “Regulations on Laboratory Animal Welfare and Ethical Examination” of China Medical University (approval number: 2017-13). General anesthesia was induced by intramuscular injection of pentobarbital (3% TIANWUD®, Tianjin, China). An additional local anesthesia was induced with 1 mL of lidocaine (2% TIANWUD®, Tianjin, China). The medial surfaces of the femur were exposed with a 2 cm incision through the skin, fascia, and periosteum parallel to the femur. A drill diameter of 2 mm was used to drill into the femur using a speeding regulating electric hand drill (Shenzhen Wincore Power Tools MZ-06, China) under profuse irrigation with sterile saline (NaCl: 9 mg mL^−1^, TIANWUD®, Tianjin, China). Two ZSM-5/Ti-2448 materials were placed on the left side, and two Ti-2448 materials were placed on the right side. The distance between implants was at least 1 cm. The tissues were sutured in separate layers and sterilized. The animals were treated with penicillin (800 thousands units per day, TIANWUD®, Tianjin, China) through intramuscular injections for three days postoperatively to prevent infection. After 4-, 12-, and 26-week postimplantation, the animals were sacrificed by intravenous injection of air under general anesthesia. Tissue samples were subjected to hematoxylin–eosin (HE) staining and immunohistochemical staining for transforming growth factor beta-1 (TGF-*β*1).

### 2.6. HE Staining

The femur containing the implants was removed, fixed by 4% neutral buffered formaldehyde, decalcified by an Ethylene Diamine Tetraacetic Acid (EDTA) solution, and embedded in paraffin. 8 *μ*m thick microtome sections were subjected to HE staining and observed using a computer-connected microscope. The estimated parameters were used to evaluate the cellular compositions around the implant materials.

### 2.7. Immunohistochemical Staining of TGF-*β*1

TGF-*β*1 belongs to the large TGF-*β* superfamily. TGF-*β*1 inhibits the osteoblast proliferation and promotes an early osteoblastic differentiation. Therefore, the expression of TGF-*β*1 can be used to evaluate the effect of early osteogenesis [14].

The paraffin was removed from the paraffin-covered sections and blocked with 3% H_2_O_2_ at room temperature for endogenous peroxidase ablation for 30 min, followed by washing with distilled water and immersion in phosphate-buffered saline (PBS) for 5 min. Antigen retrieval was performed by a microwave treatment in a sodium citrate solution, followed by cooling in ambient air. Subsequently, the paraffin sections were rinsed three times for 5 min (3 × 5 min) in PBS. TGF-*β*1 primary antibodies were diluted to a ratio of 1 : 100 and incubated for 24 h at 4°C. After triple washing with PBS, the sections were transferred to the secondary antibody for 30 min at room temperature. The sections were washed with PBS (3 × 5 min) and incubated with streptavidin-peroxidase for 30 min at 37°C. After an intensive washing (3 × 5 min with PBS), the sections were stained with 3, 3-diaminobenzidin. The process was finished by coloration with distilled water, followed by dehydration, clearing, and mounting with a resin adhesive. The average gray scale was measured using the Image-Pro Plus 6.0 software.

### 2.8. Statistical Analysis

The values were expressed as mean ± standard deviation. The Student *t*-test was used to discern significant differences between groups. The analyses were performed using the SPSS 13.0 software package. The statistical significance was set at *P* < 0.05.

## 3. Results

### 3.1. Surface Properties

**Figure 1:**
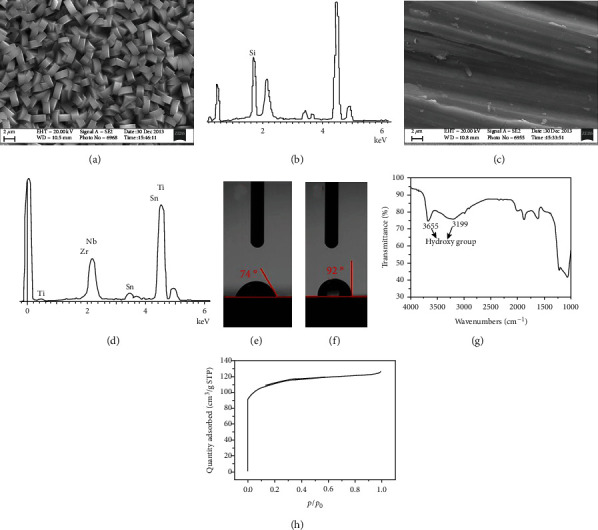
Surface characteristics of the ZSM-5/Ti-2448, Ti-2448, and ZSM-5 powders: (a, c) SEM morphologies of ZSM-5/Ti-2448 and Ti-2448; (b, d) EDS spectra of ZSM-5/Ti-2448 and Ti-2448; (e, f) contact angles of ZSM-5/Ti-2448 and Ti-2448; (g) FT-IR spectrum of the ZSM-5 powder; (h) N_2_ absorption/desorption isotherm of the ZSM-5 powder.

The SEM imaging of the bare Ti-2448 showed a relatively smooth surface with a low roughness compared to the structure of ZSM-5/Ti-2448 (Figures 1(a) and 1(c)). The chemical composition of ZSM-5/Ti-2448 was O and Si, while that of Ti-2448 was Ti, Nb, Zr, and Sn, which were observed by EDS (Figures 1(b) and 1(d)). The contact angle of ZSM-5/Ti-2448 was smaller than that of Ti-2448 (74° vs. 92°) (Figures 1(e) and 1(f)). To study the chemical properties of ZSM-5/Ti-2448, FT-IR spectroscopy was used to evaluate the ZSM-5 powder. The peaks at 3199 and 3655 in the spectrum indicate silanol groups on the surface of ZSM-5 (Figure 1(g)). The texture properties of the ZSM-5 powders were evaluated by nitrogen adsorption/desorption. The ZSM-5 powder had large specific surface area and micropore volume (Table 1, Figure 1(h)).

### 3.2. Evaluation of the Biomineralization

**Figure 2:**
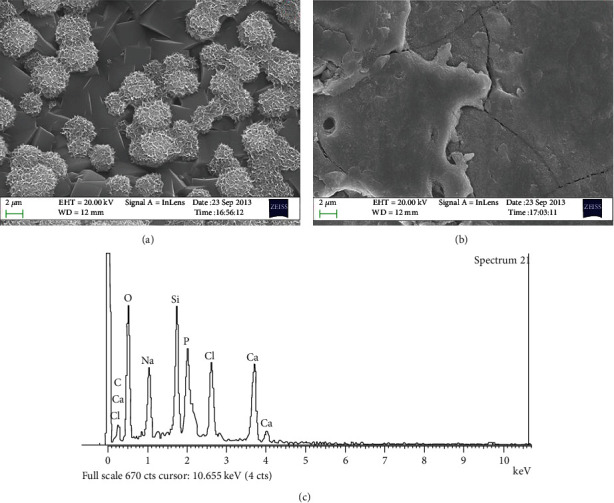
Mineralization on ZSM-5/Ti-2448 (a) and Ti-2448 (b) and EDS spectrum of the deposits on ZSM-5/Ti-2448 (c).

Deposits could be observed on the surface of ZSM-5/Ti-2448 at 14 days after incubation, while no deposits were identified on Ti-2448 (Figure 2). The chemical compositions of the deposits were O, Na, Si, Ca, P, and Cl. These deposits correspond to an apatite-like layer.

### 3.3. Cell Proliferation

**Figure 3:**
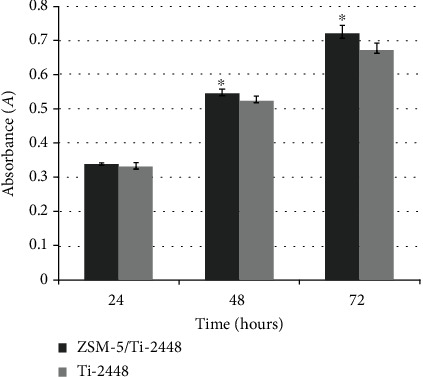
MC3T3-E1 cell proliferation of ZSM-5/Ti-2448 and Ti-2448 for 24, 48, and 72 h. The symbols indicate a statistical difference with *P* < 0.05 between the samples.

The MC3T3-E1 cell proliferation indicated by the MTT assay continuously increased with the culture time in both specimens. Despite the absence of statistical differences, the absorbance (*A*) of Ti-2448 was inferior to that of ZSM-5/Ti-2448 after 24 h of culturing (*P* > 0.05). After 48 and 72 h of culturing, significant differences were detected between ZSM-5/Ti-2448 and Ti-2448 (*P* < 0.05) (Figure 3).

### 3.4. Cell Cytotoxicity

The MTT assays showed that ZSM-5/Ti-2448 was not cytotoxic, whereas the positive control was markedly cytotoxic (Table 2).

### 3.5. Cell Differentiation

**Figure 4:**
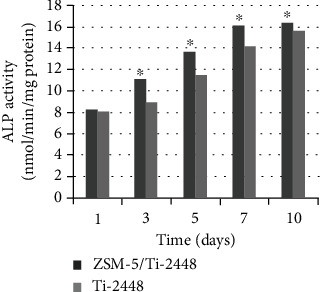
ALP activity normalized to the protein content of MC3T3-E1 cells cultured on the ZSM-5/Ti-2448 and Ti-2448 surfaces for 1, 3, 5, 7, and 10 days. The symbols indicate a statistical difference with *P* < 0.05 between the samples.

The osteoblast differentiation was assessed by measuring the ALP activity, normalized to the total protein content. The ALP activity increased with the culture time, regardless of the substrate. After one day of culturing, the ALP activity was slightly higher in the ZSM-5/Ti-2448 group than in the Ti-2448 group. However, the difference was not statistically significant (*P* > 0.05). After 3, 5, 7, and 10 days of culturing, the ALP activity in the ZSM-5/Ti-2448 group was significantly higher than that in the Ti-2448 group (*P* < 0.05) (Figure 4).

### 3.6. Endosseous Implantation

#### 3.6.1. HE Staining

**Figure 5:**
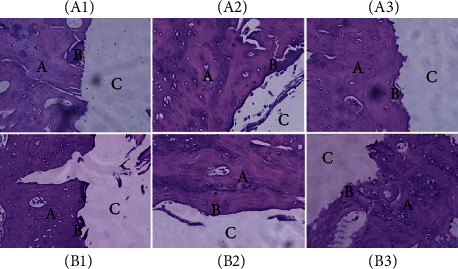
Histological observation of each group with HE staining. Few inflammatory cells are observed around ZSM-5/Ti-2448 and Ti-2448 after 4, 12, and 26 weeks of healing (20x). (A1) 4 weeks, ZSM-5/Ti-2448; (A2) 12 weeks, ZSM-5/Ti-2448; (A3) 26 weeks, ZSM-5/Ti-2448. (B1) 4 weeks, Ti-2448; (B2) 12 weeks, Ti-2448; (B3) 26 weeks, Ti-2448. A: host bone; B: new bone; C: implant material.

Notably, no signs of infection around the implants were observed in the evaluated rabbits, which demonstrates that the postoperative procedure was uneventful. Inflammatory cells around ZSM-5/Ti-2448 and Ti-2448 were stained at 4, 12, and 26 weeks after implantation (Figure 5). Few inflammatory cells were observed around ZSM-5/Ti-2448 and Ti-2448 at 4, 12, and 26 weeks after implantation. Notably, no significant difference was observed between the two materials.

#### 3.6.2. Immunohistochemical Staining of TGF-*β*1

**Figure 6:**
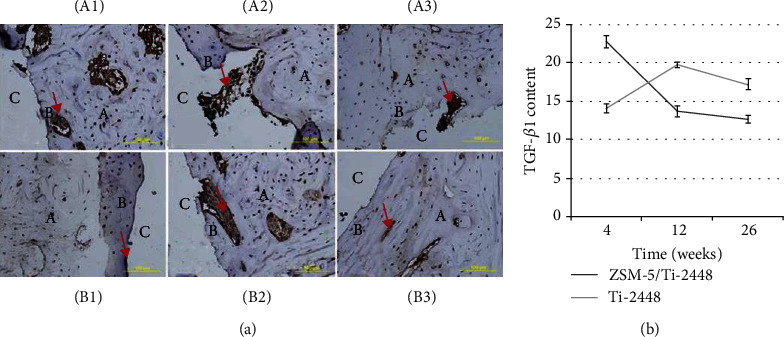
TGF-1*β* immunohistochemical staining of ZSM-5/Ti-2448 and Ti-2448 at 4, 12, and 26 weeks after implantation. (a) Photomicrograph of the immunohistochemical staining of TGF-*β*1 (20x). (A1) 4 weeks, ZSM-5/Ti-2448; (A2) 12 weeks, ZSM-5/Ti-2448; (A3) 26 weeks, ZSM-5/Ti-2448. (B1) 4 weeks, Ti-2448; (B2) 12 weeks, Ti-2448; (B3) 26 weeks, Ti-2448. A host bone, B new bone, and C implant material. The arrow shows the immunohistochemical staining of TGF-*β*1. (b) TGF-*β*1 contents of ZSM-5/Ti-2448 and Ti-2448 at 4, 12, and 26 weeks after implantation.

The expression of TGF-*β*1 was evaluated by immunohistochemical staining (Figure 6). The amount of TGF-*β*1 in the ZSM-5/Ti-2448 group was significantly higher than that in the Ti-2448 group after 4-day implantation (*P* < 0.05). And the amount of TGF-*β*1 in the ZSM-5/Ti-2448 group decreased with the implantation time. On the contrary, the amount of TGF-*β*1 in Ti-2448 was significantly higher at 12 weeks after implantation than that at 4 and 26 weeks (*P* < 0.05).

## 4. Discussion

In our study, we formed a ZSM-5 coating on Ti-2448 surface by hydrothermal synthesis. The hydrothermal synthesis method could produce a uniform coating on various shapes, which can also strengthen the adhesion between the coating and substrate owing to its chemical bond. ZSM-5/Ti-2448 had a good compatibility without cytotoxicity *in vitro* and less inflammation around the implants *in vivo*. In addition, ZSM-5 and Ti-2448 could enhance the osteoblast proliferation and differentiation. The number of osteoblasts on ZSM-5/Ti-2448 was significantly higher than that on untreated surfaces. ALP and TGF-*β*1 served as early markers of osteoblast differentiation. The ALP activity of osteoblasts on ZSM-5/Ti-2448 was significantly higher than that on Ti-2448. TGF-*β*1 peaked at 4 weeks after the implantation in the ZSM-5/Ti-2448 group and decreased with time, whereas the expression of TGF-*β*1 on the Ti-2448 samples was relatively weak at 4 weeks after the implantation, peaked at 12 weeks after the implantation, and then gradually weakened. The peak of ZSM-5/Ti-2448 occurred earlier than that of Ti-2448. Biomineralization is used to assess whether a biomaterial is ideal for bone tissue bonding and formation. A bone-like apatite layer could be formed on the surface of ZSM-5/Ti-2448 after 14 days of incubation in SBF. Thus, ZSM-5/Ti-2448 could accelerate the bone repair at an early stage.

The above results could be illustrated by the surface morphology of ZSM-5, which had a rougher surface than that of the uncoated Ti-2448. This is consistent with other studies showing that a relative roughness profile improves the cell proliferation and eventually the osseointegration of an artificial bone material [15–17]. The number of osteoblasts in the leaching liquor of materials and ALP-specific activity increased, which could also be attributed to the surface roughness [18, 19]. The porous structures of ZSM-5 could provide a larger specific surface area and pore volume, which adsorbed proteins considerably more than Ti-2448 [20, 21]. In addition, zeolite exhibits a strong dispersion force [22]. The dispersion force between molecules arises when an instantaneous dipole moment in one molecule induces a dipole moment in the second molecule, providing a good adsorption capability [23]. This adsorption capability of ZSM-5/Ti-2448 could enhance the osteoblast proliferation and differentiation.

Moreover, the ZSM-5 coating is a silica-containing material. The silicon hydroxyl group was observed on the surface of the ZSM-5 powder in the infrared spectrum, which is a hydrophilic center associated with a tetrahedrally coordinated aluminum in the zeolite framework. ZSM-5/Ti-2448 had a better hydrophilicity because of the silanol groups. Hydrophilicity is speculated to be closely involved in bone formation and adhesion of cells to materials [24]. Contact angle measurement is a useful method for the determination of the surface hydrophilicity. The contact angle of ZSM-5/Ti-2448 was approximately 74°. ZSM-5/Ti-2448 was hydrophilic with a contact angle below 90°. Schwarz and Milne [25] reported that Si deficiency results in an abnormal bone formation. The presence of Si contributes to an enhanced human primary osteoblast mineralization *in vitro* [26] and improves the mineralization rate and maturity of the newly formed bone around the artificial bone material *in vivo* [27].

## 5. Conclusion

The ZSM-5 coating modified on the Ti-2448 surface by a hydrothermal synthesis method (ZSM-5/Ti-2448) had a good biocompatibility and enhanced early osteogenesis compared to the noncoated Ti-2448. However, further studies are needed to confirm these possibilities for clinical use.

## Figures and Tables

**Table 1:** Summary of the textural properties of the ZSM-5 powder.

Sample	*S*_BET_ (m^2^g^−1^)	*S*_micro_ (m^2^g^−1^)	*S*_BJH_ (m^2^g^−1^)	*V*_micro_ (cm^3^g^−1^)	*V*_meso_ (cm^3^g^−1^)	*V*_t_ (cm^3^g^−1^)
ZSM-5/Ti-2448	417	402	15	0.173	0.024	0.197

**Table 2:** Cytotoxic test results obtained by MTT assays. Negative control: Ti-2448; positive control: 0.64% phenol solution.

Sample	RGR (%)			Cytotoxic
	24 h	48 h	72 h	
ZSM-5/Ti-2448	101.20	103.98	107.10	Not cytotoxic
Ti-2448	100	100	100	
0.64% phenol solution	22.31	15.28	12.83	Markedly cytotoxic

## Data Availability

The data used to support the findings of this study are available from the corresponding author upon request.
